# Small Cell Lung Cancer: A New Era Is Beginning?

**DOI:** 10.3390/cancers13112646

**Published:** 2021-05-28

**Authors:** Alessandro Morabito, Christian Rolfo

**Affiliations:** 1 Medical Oncology, Thoracic Department, Istituto Nazionale Tumori “Fondazione G. Pascale”-IRCCS, 80131 Napoli, Italy; 2 Center for Thoracic Oncology, Tisch Cancer Institute, Mount Sinai System & Icahn School of Medicine, Mount Sinai, New York, NY 10128, USA

Small cell lung cancer (SCLC) accounts for about 15% of all lung cancers and it is the most aggressive one. Treatment of SCLC has always represented a significant challenge for oncologists. Attempts to improve the results of first-line treatment have all failed for decades, emphasizing the need for novel therapeutic strategies and the development of validated biomarkers [1]. This scenario has only begun to change recently thanks to the overall survival advantage reached with the addition of immune checkpoint inhibitors (ICI) to first-line chemotherapy, the availability of new effective agents in pretreated patients, and improvements in the knowledge of the biology of SCLC. This Special Issue includes nine articles (one original article and eight reviews), mainly focused on the major progress in SCLC treatment, presented by international leaders in the field of thoracic oncology. The review by Raso MG et al. highlighted current pathological concepts, including classification, immunohistochemistry features, and differential diagnosis [2]. Moreover, they summarized the knowledge of the immune tumor microenvironment, tumor heterogeneity, and genetic variations of SCLC. However, the current classification of SCLC as a single entity hinders effective targeted therapies against this heterogeneous neoplasm. Recent comprehensive genomic analyses have improved our understanding of the diverse biological processes that occur in this tumor type, suggesting that a new era of molecular-driven treatment decisions is finally foreseeable for SCLC patients. A new classification based on RNA expression in mouse-derived SCLC lines has been proposed [3]. This classification identifies four main subdivisions based on the level of expressions of ASCL1 (achaete-scute homolog 1), classified as SCLC-A; NEUROD1 (neurogenic differentiation factor one), classified as SCLC-N; POU2F3 (pou class 2 homeobox 3), classified as SCLC-P; and YAP1 (yes-associated protein 1), classified as SCLC-Y. These findings highlight the heterogeneity of SCLC, with the identification of unique subtypes that could allow the deployment of targeted treatments. For patients with limited stage (LS)-SCLC, the review by Martucci N. et al. summarized the main results observed with surgery, as part of a multimodality treatment [4]. In particular, they showed that several prospective, retrospective, and population-based studies have demonstrated the feasibility of a multimodality approach, including surgery in addition to chemotherapy and radiotherapy in selected patients with stage I SCLC. For patients with extended stage (ES)-SCLC, the review by Lazzari C et al. summarized the main progress of recent years, mainly due to the introduction of immune checkpoint inhibitors, and they discussed the future directions of the clinical research [5]. Currently, the combinations of platinum and etoposide, plus atezolizumab or durvalumab, have been approved for the first-line treatment of ES-SCLC; however, there is no head-to-head comparison of these regimens with different ICIs. A systematic review and a network meta-analysis presented by Chen HL et al. firstly proposed a ranking for progression-free survival, overall survival, objective response rate, and grade 3–4 adverse events for the different combinations of ICIs and chemotherapy for ES-SCLC first-line treatment [6]. In particular, they showed that nivolumab was associated with the best ranking for overall survival, followed by atezolizumab, durvalumab, pembrolizumab, and ipilimumab, but it had also the highest probability of grade 3–4 adverse events. Of course, additional randomized phase III studies are needed to verify these conclusions. Other immunotherapy strategies are also currently being explored, including chimeric antigen receptor (CAR) T cells, tumor vaccines, antibody-drug conjugates (ADCs), and immunomodulators [7]. Moreover, preclinical studies have highlighted a consistent and complex cross-talk between immune cells and angiogenic molecules; on these bases, several clinical trials are currently ongoing to evaluate the efficacy of immunotherapy plus antiangiogenic agents in SCLC patients [8]. In the second-line setting, lurbinectedin, an oncogenic transcription inhibitor analogue of trabectedin, received accelerated approval by the FDA in early 2020 after demonstrating a favorable response rate and a duration of response in an open-label phase II trial [9]. Among the new agents in development, PARP inhibitors represent a therapeutic class that has become an important treatment option for multiple tumor types, although their single agent activity in SCLC is limited [10]. Combining PARP inhibitors with agents that damage DNA and inhibit DNA damage response (DDR), as well as enhancing antitumor immunity down-stream of DNA damage, represent rational therapeutic strategies with preclinical and early trial data to support specific combinations. Finally, the high aggressiveness of SCLC and the lack of active treatments underlie the need for the identification of biomarkers that can aid in the development of personalized medicine in SCLC. Non-invasive biomarkers in peripheral blood, including circulating tumor cells (CTCs) or cell free DNA (cfDNA), can offer the opportunity to achieve prognostic and/or predictive information to study mechanisms of resistance and discover novel targets for therapeutic approaches, thus overcoming the frequent inadequate amounts of tumor samples [11]. A European pooled analysis of 367 individual patients’ data confirmed that higher pre-treatment CTC counts are a negative independent prognostic factor in SCLC when considered as a continuous variable or as dichotomized counts of ≥15 or ≥50 [12]. Therefore, incorporating CTC counts as a continuous variable could improve clinical–pathological prognostic models. In addition, the analysis of the molecular profile of CTCs and the generation of CTC-derived xenografts (CDXs) are encouraging deeper knowledge of SCLC biology, with the major finding that SCLC is a highly heterogeneous disease. In conclusion, is a new era beginning for SCLC? We believe so. We hope that the current lack of targetable oncogenic drivers for SCLC will be overcome by the application of novel technologies of molecular analysis, the identification of different molecular subtypes, and the definition of molecular markers which are predictive of a response to new targeted agents, thus allowing significant advances in the knowledge of SCLC and a better customization of the treatments for each patient.
